# Comparing key characteristics of young adult crack users in and out-of-treatment in Rio de Janeiro, Brazil

**DOI:** 10.1186/1747-597X-9-2

**Published:** 2014-01-10

**Authors:** Marcelo Cruz, Neilane Bertoni, Francisco I Bastos, Chantal Burnett, Jenna Gooch, Benedikt Fischer

**Affiliations:** 1 Institute of Psychiatry, Federal University of Rio de Janeiro, Av. Venceslau Bras, 71 Fundos, Rio de Janeiro 22290-140, Brazil; 2 Institute of Communication and Scientific Information & Technology for Health, Oswaldo Cruz Foundation, Av. Brazil, 4365 – Manguinhos, Rio de Janeiro 21040-360, Brazil; 3 Centre for Applied Research in Mental Health and Addiction (CARMHA), Faculty of Health Sciences, Simon Fraser University, 2400-515 West Hastings St., Vancouver V6B 5 K3, Canada; 4 Social & Epidemiological Research, Centre for Addiction and Mental Health, 33 Russell St., Toronto M6J 1H4, Canada; 5 Department of Psychiatry, University of Toronto, 250 College Street, Toronto M5T 1R8, Canada

**Keywords:** Brazil, Crack use, Health characteristics, Marginalization, Treatment access, Service utilization

## Abstract

**Background:**

Crack use is prevalent among street drug users in Brazilian cities, yet despite recent help system reforms and investments, treatment utilization is low. Other studies have identified a variety of – often inconsistent – factors associated with treatment status among crack or other drug users. This study compared socio-economic, drug use, health and service use characteristics between samples of young adult crack users in- and out-of-treatment in Rio de Janeiro, Brazil.

**Findings:**

Street-involved crack users (n = 81) were recruited by community-based methods, and privately assessed by way of an anonymous interviewer-administered questionnaire as well as biological methods, following informed consent. In-treatment users (n = 30) were recruited from a public service in-patient treatment facility and assessed based on the same protocol. Key indicators of interest were statistically cross-compared. Not-in-treatment users were less likely to: be white, educated, stably housed, to be involved in drug dealing, to report lifetime marijuana and current alcohol use, to report low mental health status and general health or addiction/mental health care; they were more likely to: be involved in begging and utilize social services, compared to the in-treatment sample (statistical significance for differences set at p < .05).

**Conclusions:**

In-treatment and not-in-treatment crack users differed on several key characteristics. Overall, in-treatment users appeared to be more socio-economically integrated and connected to the health system, yet not acutely needier in terms of health or drug problems. Given overall low treatment utilization but high need, efforts are required to facilitate improved treatment access and use for marginalized crack users in Brazil.

## Introduction

Crack use is prevalent and entails extensive health and social problems in Brazil. There may be up to 1 million crack users in Brazil [1,2]; most are young, poor, socio-economically marginalized and crime-involved [3]. Most feature extensive poly-substance use (including alcohol, marijuana, stimulants/inhalants), yet do not engage in injection drug use (IDU); hence, blood borne virus (BBV), e.g., HIV or Hepatitis C Virus (HCV), rates are comparably low among Brazilian crack users [4,5]. Violence, e.g., related to drug markets, contributes to high levels of injury and mortality among users, and social problems in many urban communities in Brazil [6,7].

While care (including treatment) services for substance use problems have been expanded in Brazil in recent years, their utilization – specifically by crack users – appears rather limited [8,9]. However, it is unclear whether these patterns are mainly determined by limitations in service availability, or access barriers between services and the (marginalized) user populations [9].

Studies examining differences between crack/cocaine as well as opioid/heroin user populations in and out-of-treatment have predominantly found the latter sub-groups to be more commonly characterized by social marginalization (e.g., unstable housing, unemployment) and limited social supports; lengthier, more complex and/or problematic drug use histories or patterns; more intensive (physical and/or mental) health problems; and higher crime or criminal justice involvement; however, findings are heterogeneous and inconsistent [10-13]. While comparative Brazilian studies are scarce, Ferri et al. found higher levels of homelessness, sex trade, crime and problematic substance use among out-of-treatment compared to in-treatment cocaine users in Sao Paulo [14]. Malta et al. [8] found socio-demographic factors (race, education, employment) to be associated with treatment-seeking among impoverished drug users in Rio de Janeiro (RdJ).

The purpose of this study was to compare key socio-economic, drug use and health characteristics of two samples of young adult in- and out-of-treatment crack users in RdJ, Brazil; given the acute extent of crack use in Brazil yet low treatment utilization, this comparative analysis aids to identify potential factors differentiating treatment status, and to inform interventions towards improving treatment access and/or utilization.

## Findings

### Methods

The study conducted a comparative, cross-sectional assessment of two community-recruited convenience samples of young out-of-treatment and in-treatment crack users. Eligibility criteria for both samples were: 1) Crack use on 3+ days/week in last 3 months (for in-treatment participants, this criterion applied to pre-treatment entry); 2) 18–24 years of age; 3) ability to consent to study protocol, facilitating basic comparability of retrospective data on these grounds. Street-involved users were recruited by way of community-based outreach methods in a poor neighborhood (Jazarezinho) of RdJ known for street drug use. Local community contacts distributed key study information among users, and prospective participants underwent a brief in-person eligibility assessment. The treatment sample was recruited from among in-patients in a public drug treatment clinic (capacity: 90 beds; average program duration 30–45 days, with most admissions by self- or family-referral) in RdJ. Similarly, potentially eligible patient participants recruited in the clinic were informed about the study by clinic staff in the clinic setting, then contacted study staff if they expressed interest and were subsequently screened for eligibility. The locale from which street users were recruited and the clinic are situated within different parts of RdJ. The clinic is the only general and public referral clinic in RdJ for in-patient drug abuse treatment mainly catering to poor and marginalized users. While the clinic’s crack user patients are referred from different neighborhoods across the city, including the one where street users were recruited, the general profile of the clinic’s patient population allows to reasonably assume the inclusion of overall similar and comparable study populations.

Upon eligibility confirmation and provision of informed consent, participants were assessed via an interviewer-administered, anonymous questionnaire comprising social, drug use, and health characteristics; furthermore, blood samples for anonymous BBV testing were collected. Assessments took about 45–60 minutes, and were conducted by field research assistants trained and experienced in field research with marginalized populations. Participants received a public transportation pass for their time and efforts. The study protocol was approved by the Ethical Review Committee, Institute of Psychiatry, Federal University of Rio de Janeiro, as well as the Brazilian National Ethics Committee (CONEP 519/2010; see also [9] for additional study details).

A total of n = 81 street-involved, and n = 30 in-treatment users were assessed between November 2010 and July 2011. Data were entered into an electronic database; descriptive statistics on relevant outcome indicators were computed with SPSS. Specifically, we computed proportions for categorical variables, and means for continuous variables, including 95% Confidence Intervals (CI), and statistically compared these indicators between the two groups by way of chi-square and t-tests, respectively, with significance levels set at p < .05.

### Results

The mean age in both samples was 21 years (range 18–24; SD: 2; data not shown). Respective majorities in both samples were male; single or separated; unemployed and had been arrested; a minority in both groups engaged in sex work. The street sample: included fewer white people; had lower education; was more likely to be unstably housed and to be involved in begging – but less likely to be involved in drug dealing – than the treatment sample (Table 1).

**Table 1:** Socio-demographic and -economic characteristics of samples

	**Street sample (n = 81)**			**Treatment sample (n = 30)**		
	**n**	**%**	**95% CI**	**n**	**%**	**95% CI**
**Sex**						
Male	54	67	57–77	23	79	64–94
Female	26	33	23–43	6	21	6–36
Colour/Race [*]						
White	8	10	4–17	8	27	11–43
Non-white	73	90	84–97	22	73	57–89
**Marital status**						
Single or separated	70	86	79–94	24	80	66–94
Married or co-habitating	11	14	6–22	6	20	6–34
Education [*]						
No formal education or some elementary school	69	86	79–94	14	47	29–65
Completed elementary school or higher	11	14	6–22	16	53	35–71
Housing status [30] [*]						
Stable	20	25	16–35	27	90	80–101
Unstable (including homelessness)	60	75	66–85	3	10	0–21
**Employment status [30]**						
Employed or working	33	41	30–52	15	50	32–68
Not employed	48	59	48–70	15	50	32–68
**Arrested (in past year)**						
Yes	23	28	18–38	12	40	23–58
No	58	72	62–82	18	60	43–76
Drug dealing for income [30] [*]						
Yes	7	9	3–15	8	27	11–43
No	74	91	75–91	22	73	57–89
**Sex work for income [30]**						
Yes	14	17	9–25	3	10	0–21
No	67	83	75–91	27	90	79–101
Begging for income [30] [*]						
Yes	20	25	16–34	1	3	0–9
No	61	75	66–84	29	97	91–103

Notes:

[30]: In past 30 days.

[*] Chi-square significant at p < 0.05 level.

Both samples, on average, had a history of about 4 years of crack use as well as reported between 10 and 12 crack use episodes per day (data not shown). None except one participant had an IDU history. A larger proportion of non-treatment participants shared crack pipe implements. Approximately half or more participants in both samples reported lifetime and current use of alcohol, tobacco, marijuana and cocaine. Larger proportions in the treatment sample reported current (i.e., past 30 days) alcohol use (Table 2).

**Table 2:** Crack and other drug use characteristics of samples

	**Street sample (n = 81)**			**Treatment sample (n = 30)**		
	**N**	**%**	**95% CI**	**n**	**%**	**95% CI**
Shared crack implements [30] [*]						
Yes	49	60	49–71	8	27	11–43
No	32	40	29–51	22	73	57–89
**Shared >10 time (among sharers)**						
Yes	33	67	54–80	3	38	4–72
No	16	33	20–46	5	62	28–96
**Drug injection history (ever)**						
Yes	0	n/a	n/a	1	3	n/a
No	81	100	n/a	29	97	91–100
**Use of other drugs [30]**						
Alcohol [*]	21	34	22–46	13	65	43–85
Tobacco	70	92	86–98	25	96	89–100
Cocaine	14	26	14–38	9	45	23–67
Marijuana	42	64	52–76	19	66	49–83
Benzodiazepines	0	n/a	n/a	1	33	n/a
Inhalants (e.g., glue, solvents)	2	5	0–12	2	20	0–43

Notes:

[30]: In past 30 days.

[*] Chi-square significant at p < 0.05 level.

About half the participants in both samples reported physical health to be ‘good’ or better, and that they had some physical health problems. While about a third in each group reported mental health problems, fewer – about half – of in-treatment participants rated their mental health to be ‘good’ or better. A majority in both samples reported unsafe sex, while only a minority had ever been HIV tested. Small proportions in both groups were HBV (antigen) positive; a small proportion in the street group was HIV-positive. Minorities in each group reported general health, specialized addiction/mental health, and social service utilization; social service utilization was higher among non-treatment users; general and specialized health service utilization was higher among in-treatment users (Table 3).

**Table 3:** Key health and service utilization indicators of samples

	**Street sample (n = 81)**			**Treatment sample (n = 30)**		
	**n**	**%**	**95% CI**	**n**	**%**	**95% CI**
**Self-rated physical health status [30]**						
Excellent, very good, or good	43	53	42–64	13	43	25–61
Fair or poor	38	47	36–58	17	57	39–75
**Physical health problems [30]**						
Yes	32	41	30–52	12	41	23–59
No	47	59	48–70	17	59	41–77
Self-rated mental health status [30] [*]						
Excellent, very good, or good	45	56	45–67	9	31	14–48
Fair or poor	35	44	33–54	20	69	52–86
**Mental health problems [30]**						
Yes	30	37	27–48	11	37	20–54
No	51	63	53–74	19	63	46–80
**Unprotected sex [30]**						
Yes	45	56	45–67	22	73	57–89
No	36	44	33–55	8	27	11–42
**Tested for HIV (ever)**						
Yes	34	42	31–53	11	37	20–54
No	46	58	47–69	19	63	46–80
**HIV + (serology)**	3	4	0–8	0	n/a	n/a
**Hep B + (HBSAg)**	5	6	1–11	0	n/a	n/a
**Hep C + (HCVAB)**	0	n/a	n/a	0	n/a	n/a
Social services use (e.g., shelter, food bank) [30] [*]						
Yes	27	33	23–43	4	13	1–25
No	54	67	57–77	26	87	75–99
General health services use (e.g., community health centre, hospital) [30] [*]						
Yes	8	10	4–17	9	30	14–46
No	73	90	84–97	21	70	54–86
Specialized mental health or addiction services use [30] [*]						
Yes	0	n/a	n/a	3	10	0 - 21
No	69	100	n/a	26	90	79 - 101

Notes:

[30]: In past 30 days.

[*] Chi-square significant at p < 0.05 level.

## Discussion

We found notable differences in key characteristics between the non-treatment and treatment samples of crack users, several of which mirror findings from other studies. For example, lower rates of visible minorities (e.g., non-whites) and higher education status have been found among in-treatment samples elsewhere, suggesting that socio-economic status may play a role in treatment seeking or access; this could relate to the access of relevant information, the management of bureaucratic systems or simply monetary resources [8,12,13]. Housing status is recognized as a primary determinant of health, and specifically for risk and harm outcomes among drug users [15,16]. Thus, it may act as a form of ‘social capital’ facilitating the dynamics of treatment access [10,12,14]. While less intensely crime-involved individuals are generally more likely to access treatment, our treatment sample indicated more prolific involvement in drug dealing (but less begging) [14]. There is no readily evident explanation for this difference, also since the treatment program under study did not include compulsory treatment referrals.

While the two groups showed similar crack and IDU patterns, the non-treatment group featured a higher prevalence of crack implement sharing – a risk behavior that possibly facilitates BBV (e.g., HCV) transmission [17]. This mirrors other studies’ findings that key risk behaviors (e.g., needle sharing) are more commonly found among socio-economically marginalized (e.g., homeless) drug users [16,18]. The groups featured similar profiles of other current (non-crack) drug use, with the exception of alcohol use which was more prevalent among the in-treatment group. Since the intensity of drug use patterns or problems can influence treatment-seeking [19], alcohol-related problems may have been a factor in treatment-seeking in the in-treatment group; however, we do not have evidence for other, similar drug use-related differentiating factors which hence likely need to be identified in other individual or ecological domains.

Users’ physical and mental health status are associated with treatment access and status [20,21]. Our study’s in-treatment group indicated lower mental health status self-ratings which – while no differences were found for mental health problems – may directly relate to the severity of crack or other drug use related problems (e.g., withdrawal) experienced, or otherwise motivated treatment seeking. We found important differences regarding health and social service utilization patterns. While any service utilization was limited to respective sample minorities, higher social service utilization among non-treatment participants may suggest higher need for and reliance on ‘survival’ services (e.g., food-banks, shelter), consistent with their higher socio-economic marginalization. Conversely, the in-treatment group appeared to be better connected with health services, which may be a factor facilitating treatment access.

While extensive treatment needs for crack use appear to exist, treatment service access and utilization have been found to be distinctly low in Brazil (e.g., [8,9]). In-treatment users in this study were less socio-economically marginalized (e.g., regarding education, housing) and more connected to the service system. These factors have been recognized as important in facilitating health service utilization among drug users [22,23]. Correspondingly, the more marginalized crack users were less likely to access or utilize treatment (and other health services). This is disconcerting also since marginalized drug users typically feature more acute or severe health risks or care needs [16]. Multi-site data are needed to compare the situation, and related factors, to elsewhere in Brazil. In the context of extensive prevalence of crack use and harms in Brazil, our study’s findings may support calls for both an expansion of treatment resources at least in the specific contexts of our study, as well as for existent services to be more effectively tailored to the target population; these are supported by recent related analyses finding that crack users predominantly refrain from utilizing existing services yet strongly desire to access suitable treatment if available to them [9]. Substantive service expansions have recently been implemented in Brazil; for example, the number of community-based help centers designated for alcohol and drug problems (CAPS-AD) almost doubled from 1010 (2006) to 1803 (2012) [24]. These efforts, however, appear to be insufficient to date. In addition, fundamental discussions are ongoing regarding the nature and range of treatment options needed for crack use [25]. While several of the differences (e.g., social marginalization, health risks/status) we found between the samples are not easily amenable to correction by interventions, these imply that efforts to connect crack users with treatment in particular should focus on the most marginalized users and those characterized by key health risks or problems; these efforts may best occur by community- and/or peer-based based outreach or other targeted efforts.

Our study’s limitations include that it relied on data from relatively small, non-representative convenience samples, including possible selection biases, e.g. as related to sampling, which therefore cannot be generalized. Data collection utilized self-report methods (except for BBV testing) which cannot be objectively validated and social desirability dynamics may have influenced responses however, study and assessment design (including protection of participant identity and data confidentiality) and experiences from other studies have shown similar data to be valid [26]. Samples were not assessed by clinical diagnosis instruments for severity of drug problems or clinical needs (e.g., crack dependence); however, both samples fulfilled the same eligibility criteria regarding intensive crack use, and were hence comparable on these grounds. Further, our study assessed treatment participation but not treatment outcomes.

In sum, we found important differences between in- and out-of-treatment crack users in the specific context of RdJ, Brazil; these suggest a need for improved treatment service availability, access and delivery – with particular attention to the most marginalized and high-risk users – in the target population.

## Abbreviations

BBV: Blood borne virus; HBV: Hepatitis B virus; HCV: Hepatitis C virus; HIV: Human immunodeficiency virus; IDU: Injection drug use; Rdj: Rio de Janeiro; SPSS: Statistical package for the social sciences.

## Competing interests

The authors declare that they have no competing interests.

## Authors’ contributions

MC, FB, BF designed the study protocol and data analysis plan. NB managed the study data and conducted the data analysis. CB and JG conducted relevant literature searches and reviews, and integrated them with study data. BF, MC and FB jointly led the manuscript writing. All authors contributed to data interpretation and manuscript revisions, and have read and approved the final manuscript.
